# Insights into non-proteinaceous ubiquitination

**DOI:** 10.1042/BST20253029

**Published:** 2025-04-03

**Authors:** Emily L. Dearlove, Danny T. Huang

**Affiliations:** 1 Cancer Research UK Scotland Institute, Garscube Estate, Switchback Road, Glasgow G61 1BD, U.K; 2 School of Cancer Sciences, University of Glasgow, Glasgow G61 1QH, U.K.

**Keywords:** adenosine diphosphate ribose, carbohydrate, lipid, nucleic acids, ubiquitin

## Abstract

Ubiquitination plays a key role in the regulation of numerous diverse cellular functions. This process involves the covalent attachment of ubiquitin to protein substrates by a cascade of enzymes. In recent years, various non-proteinaceous substrates of ubiquitination have been discovered, expanding the potential for the functions of ubiquitination beyond its conventional role as a post-translational modification. Here, we profile the non-proteinaceous substrates of ubiquitination reported to date, the enzymes that regulate these activities, and the mechanistic details of substrate modification. The biological functions linked to these modifications are discussed, and finally, we highlight the challenges hindering further progress in the identification and functional characterization of non-proteinaceous substrates of ubiquitination within cellular contexts.

## Introduction

Ubiquitination involves the covalent attachment of the 8 kDa protein ubiquitin (Ub) to a substrate. It proceeds via a cascade of three enzymes starting with an Ub-activating enzyme (E1). The E1 binds Ub and Mg^2+^-ATP, catalyzing adenylation of the C-terminal glycine of Ub and releasing pyrophosphate. Ub is then transferred to the active site cysteine of the E1, forming a thioester linkage with the release of AMP. Next, the transfer of Ub from the E1 to the active site cysteine of an Ub-conjugating enzyme (E2) occurs by a transthioesterification reaction. Finally, Ub-ligases (E3s) catalyze the transfer of Ub to the substrate. Canonically, this post-translational modification (PTM) is characterized as the conjugation between the C-terminal glycine of Ub and the ε-amino group of a lysine residue in a protein substrate, forming an isopeptide bond. In recent years, additional amino acids have been identified as points of conjugation for non-lysine ubiquitination such as the attachment of Ub to cysteine residues by thioester linkages [1] and to serine and threonine by oxyester linkages [2]. Ub can also be conjugated to the N-terminal-NH_2_ group of substrates [3,4], including Ub itself, leading to the formation of linear polyUb chains [5]. While tyrosine contains a hydroxyl group in its aromatic side chain, to date, there is no direct evidence of ubiquitination at this site. The reduced thermodynamic stability of non-canonical Ub linkages compared with the isopeptide bond generated with attachment to lysine and resulting difference in reaction kinetics initially raised questions about the physiological relevance of these modifications. However, evidence of these modifications from cells manifests potential for functions of non-lysine ubiquitination in highly dynamic systems. Beyond non-lysine ubiquitination, various non-proteinaceous substrates have emerged in recent years. These substrates include phospholipids, carbohydrates, glycolipids, metabolites, and nucleic acids. In this mini-review, we summarize the mechanistic insights into their ubiquitination and discuss the technical challenges in deciphering their roles under cellular contexts.

### Ub and Ub-like protein modification of phospholipids

**Figure 1:**
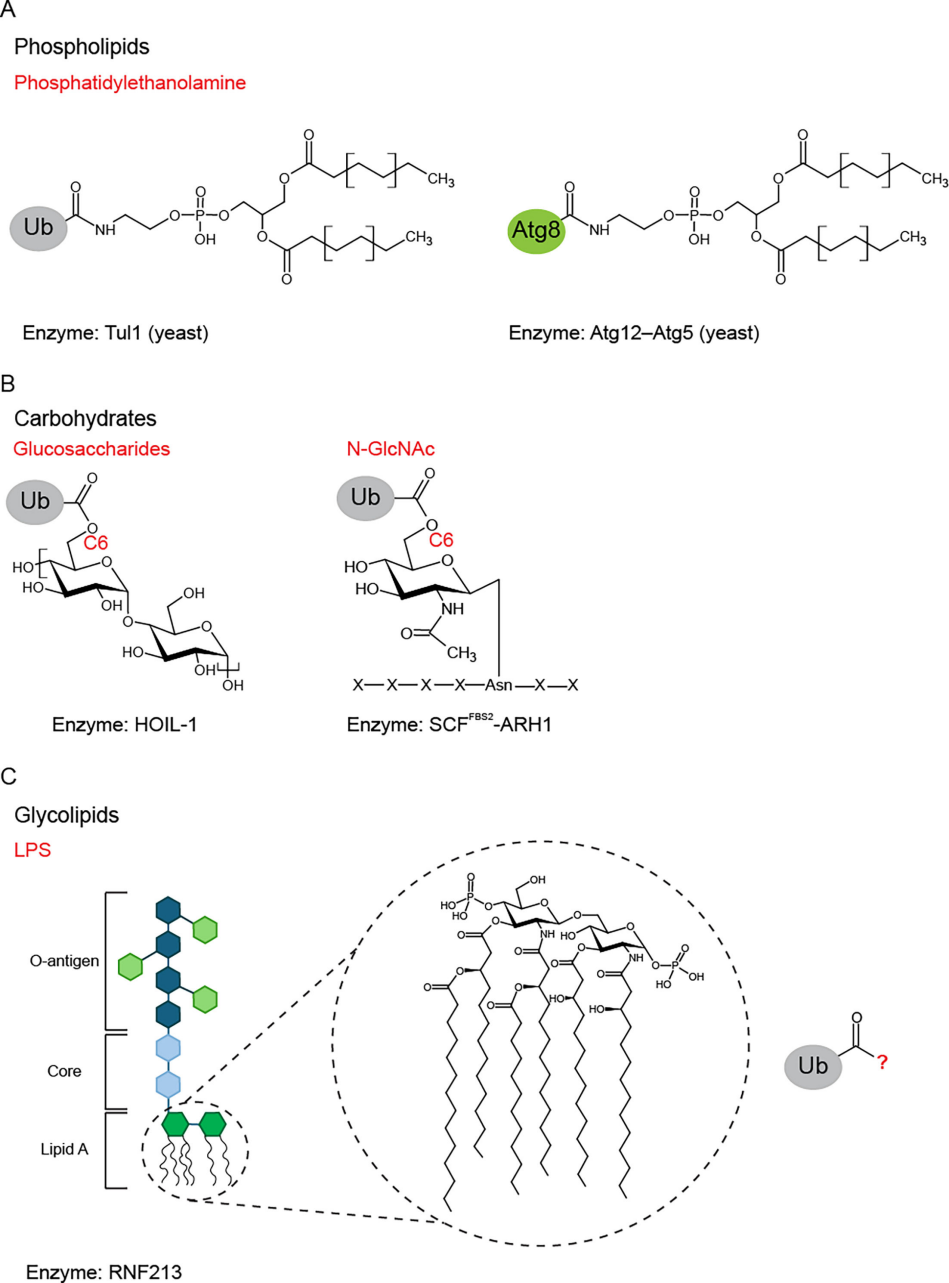
Types of non-proteinaceous substrates. (**A**) Phospholipid, phosphatidylethanolamine, is conjugated to Ub by an amide bond which in yeast is catalyzed by Tul1 (left). The E3-like complex Atg12-Atg5 catalyzes the conjugation of Atg8 to phosphatidylethanolamine by an amide bond in yeast (right). (**B**) HOIL-1 catalyzes ubiquitination of glucosaccharides including unbranched glycogen and maltoheptaose (left). The linkage between Ub and the C6 hydroxyl of glucose is an oxyester bond. N-GlcNAc (right) is ubiquitinated on the C6 hydroxyl forming an oxyester bond. This is catalyzed by the SCF^FBS2^-ARH1 complex. (**C**) Bacterial LPS is ubiquitinated by RNF213. The site of ubiquitination on Lipid A remains unknown. All chemical structures were produced using ChemSketch [7].]. HOIL-1, heme‐oxidized IRP2 ubiquitin ligase‐1; Tul1, transmembrane E3 ubiquitin-protein ligase 1; Ub, ubiquitin.

Phosphatidylethanolamines (PEs) serve as a substrate for ubiquitination. In yeast cells, the endosomal and vacuolar membranes are sites where Ub is attached to the amino group of PE via its C-terminal glycine [6] (Figure 1A). This modification is catalyzed by canonical E1, E2, and E3 enzymes, Uba1, Ubc4, and transmembrane E3 Ub-protein ligase 1 (Tul1), respectively [6]. The ubiquitination of PE is also conserved in mammals and baculoviruses. In addition to Ub modification of PEs, the conjugation of PE with Ub-like proteins (UBLs) has also been observed. Conjugation of a UBL to a non-proteinaceous substrate was first noted with the autophagy-related protein 8 (Atg8) conjugation system. Atg8 is covalently attached to PE in membranes, forming an amide bond between the head group of PE and a glycine residue in Atg8 [8] (Figure 1A). This reaction is catalyzed by a cascade of enzymes including E1-like enzyme Atg7, E2-like enzyme Atg3, and E3-like enzyme complex Atg12-Atg5 conjugate [9,10]. The Atg8-PE modification is associated with changes in membrane curvature and plays a role in the formation of autophagosomes [11]. Other UBLs, such as NEDD8 and ISG15, can also be conjugated to phospholipids in mammalian cells [6]. This broadens the scope for Ub or UBL modifications to be commonplace for phospholipids. While some cellular stressors have been identified, such as nitrogen starvation in yeast and mTOR inhibition in HeLa cells, there are still key questions surrounding the physiological function of PE modified with Ub or UBLs that are yet to be answered. As Ubc4/5 and Tul1 also ubiquitinate membrane proteins in addition to PE, it is difficult to study the specific role of Ub-PE simply by using yeast cells deficient in these enzymes. Likewise, in human cells, the enzymes responsible for ubiquitination of PE have yet to be identified. Further investigation of the biological relevance of Ub-PE remains limited in the absence of tools that modulate PE ubiquitination.

### Ub modification of carbohydrates

In addition to ubiquitinating serine and threonine residues in proteins [12], RING-between-RING (RBR) E3 ligase heme‐oxidized IRP2 ubiquitin ligase‐1 (HOIL-1) also monoubiquitinates glycogen and unbranched glucosaccharides [13]. Both of these non-lysine ubiquitination activities involve the formation of oxyester bonds. The attachment of Ub to unbranched glucosaccharides, such as maltoheptaose, occurs at the C6-hydroxyl moiety of glucose (Figure 1B). The ubiquitination of maltoheptaose is markedly increased in the presence of Met1-linked or Lys63-linked Ub chains [13]. These Ub chains were shown to bind to the in-between-RING (IBR) and preceding helical region in RBR domain to activate HOIL-1. In addition, HOIL-1 is also capable of attaching pre-assembled Ub chains to unbranched glucosaccharides [13]. It remains unclear whether HOIL-1 contains a carbohydrate-binding site. HOIL-1 is a component of the linear Ub chain assembly complex (LUBAC), comprising HOIL-1, HOIP, and SHARPIN, where HOIP catalyzes the formation of linear Met1-linked Ub chains [5,14–16]. It was shown that both SHARPIN and particularly HOIP bind specifically to the unbranched polysaccharide amylose, but not HOIL-1 [13], and it is possible that LUBAC facilitates ubiquitination of unbranched polysaccharides by HOIL-1. These findings are primarily derived from *in vitro* ubiquitination reactions. Prevention of polyglucosan deposits in various tissues in mice requires HOIL-1 E3 ligase activity [13]. While it has been hypothesized that its *in vivo* activity in preventing polyglucosan deposition and the ubiquitination of unbranched glucosaccharides *in vitro* could be linked, the connection between these two activities has yet to be established. This highlights the importance of developing tools for the identification and characterization of non-proteinaceous substrates under cellular conditions.

This is again relevant for studying asparagine-linked N-acetyl glucosamine (N-GlcNAc) ubiquitination by SCF^FBS2^-ARH1. Endo-β-N-acetylglucosaminidase catalyzes the deglycosylation of N-glycans, forming N-GlcNAc. This activity is required to generate N-GlcNAc acceptor sites on nuclear factor erythroid 2-like 1 (Nrf1) for ubiquitination by SCF^FBS2^-ARH1. These two E3 ligases act co-operatively with the ARH1-associated E2, UBE2L3, to ubiquitinate N-GlcNAc motifs at the 6-hydroxyl group by an oxyester linkage [17] (Figure 1B). Man_3_GlcNAc_2_, an oligosaccharide required for efficient FBS2 binding, is also essential for Ub modification of glycopeptides [17]. It is proposed that SCF^FBS2^ recognizes Man_3_GlcNAc_2_, while ARIH1-UBE2L3 binds NEDD8 in SCF^FBS2^ and mediates the deposition of Ub onto N-GlcNAc or Ser and Thr residues on Nrf1, which are also ubiquitinated by SCF^FBS2^-ARH1 through oxyester linkages. The ARIH1-UBE2L3 complex catalyzes the attachment of the first Ub molecule onto Nrf1, as well as the formation of polyUb chains [17]. K6, K11, K33, and K48 linkages are all present in polyUb chains on N-GlcNAc, generating highly complex heterotypic chains [17]. The SCF^FBS2^-dependent polyubiquitination of Nrf1 inhibits its activation by DDI2, providing potential physiological relevance to the modification. However, much of the characterization of the ubiquitination of N-GlcNAc comes from *in vitro* experiments using synthetic glycopeptide substrates. The presence of ubiquitinated N-GlcNAc motifs on Nrf1, the predicted endogenous substrate, has not yet been detected.

In addition to the ubiquitination of carbohydrates by E3 ligases, multiple E2s have now been identified as capable of catalyzing ubiquitination of glycerol and glucose residues, namely, UBE2J2, UBE2Q1, and UBE2Q2[18]. These E2s can attach Ub to glycerol and glucose by ester linkages. UBE2J2 and UBE2Q1 can additionally ubiquitinate the unbranched polysaccharide maltoheptaose.

### Ub modification of glycolipids

An additional class of recently identified non-proteinaceous substrate for ubiquitination is glycolipids. The RING E3 ligase, RNF213, ubiquitinates the lipid A moiety of bacterial lipopolysaccharide (LPS) on cytosol invading *Salmonella enterica serovar Typhimurium (S. Typhimurium),* forming a conjugate that is a fundamental element for the formation of the bacterial Ub coat [19] (Figure 1C). Ubiquitination of LPS, similar to its autoubiquitination activity, proceeds independently of its RING domain. Instead, it requires ATP binding by catalytically active AAA+ domains located in the dynein-like core, as well as ATP hydrolysis activity of the fourth AAA+ domain. The presence of the RZ finger is also necessary for the ubiquitination of LPS [19], specifically residue His^4509^, which is one of four evolutionarily conserved residues that coordinate Zn^2+^. This residue is required for RNF213-mediated ubiquitination of bacterial LPS [19,20] and the formation of Ub coats [20]. While the exact site of modification on lipid A remains unknown, the linkage is susceptible to degradation under alkaline conditions, indicating that it involves a hydroxyl group [19]. Whether this conjugation occurs on the sugar, fatty acid, or phosphate groups of lipid A, however, remains unclear.

The activity of LUBAC has been shown to depend on pre-existing Ub modifications on *Salmonella* [21]. RNF213-mediated ubiquitination of LPS on *Salmonella* probably serves to recruit HOIP of LUBAC, thereby linking ubiquitination of LPS to a probable physiological function. HOIP recruits LUBAC to Ub-modified *Salmonella*, enabling the attachment of Met1-linked Ub to monoubiquitinated LPS, leading to autophagy-mediated lysosomal degradation of the intracellular bacteria [21,22]. In addition, the attachment of Met-1 linked Ub to monoubiquitinated LPS induces activation of NF-κB, which promotes secretion of pro-inflammatory mediators from infected cells and restricts proliferation of cytosolic bacteria [21,22]. In addition to sensing Gram-negative *Salmonella*, RNF213 is capable of recognizing Gram-positive *Listeria monocytogenes*, the parasite *Toxoplasma gondii,* and viruses [23–27]. Ub coat formation mediated by RNF213 also occurs on *L. monocytogenes* and *T. gondii* [20], and, similar to *Salmonella*, ubiquitination of *L. monocytogenes* and *T. gondii* requires the RZ finger [20]. Mutation of His^4509^ resulted in defects in ubiquitination of *L. monocytogenes* and *T. gondii,* and mutation of Cys^4516^, a proposed catalytic residue, had similar effects in all three pathogens [20]. The ligands that are ubiquitinated on these two additional pathogens have not yet been determined, and the question remains around how RNF213 is able to recognize such a diverse range of pathogens. In simians, RNF213 faces strong positive selection particularly in its N-terminus [20]. This raises the potential for direct interactions between RNF213 and pathogen-associated molecular patterns on the pathogens it senses. A CBM20 carbohydrate-binding domain in the positively selected N-terminus has been identified [20], though ambiguous results surrounding the importance of this region in the recruitment of RNF213 mean that further investigation into this domain is required to elucidate its function. While evidence linking the ubiquitination of this non-proteinaceous substrate to a physiological function is convincing, the finer details concerning the mechanism and precise site of modification are still lacking.

### Ub modification of adenosine diphosphate ribose

**Figure 2:**
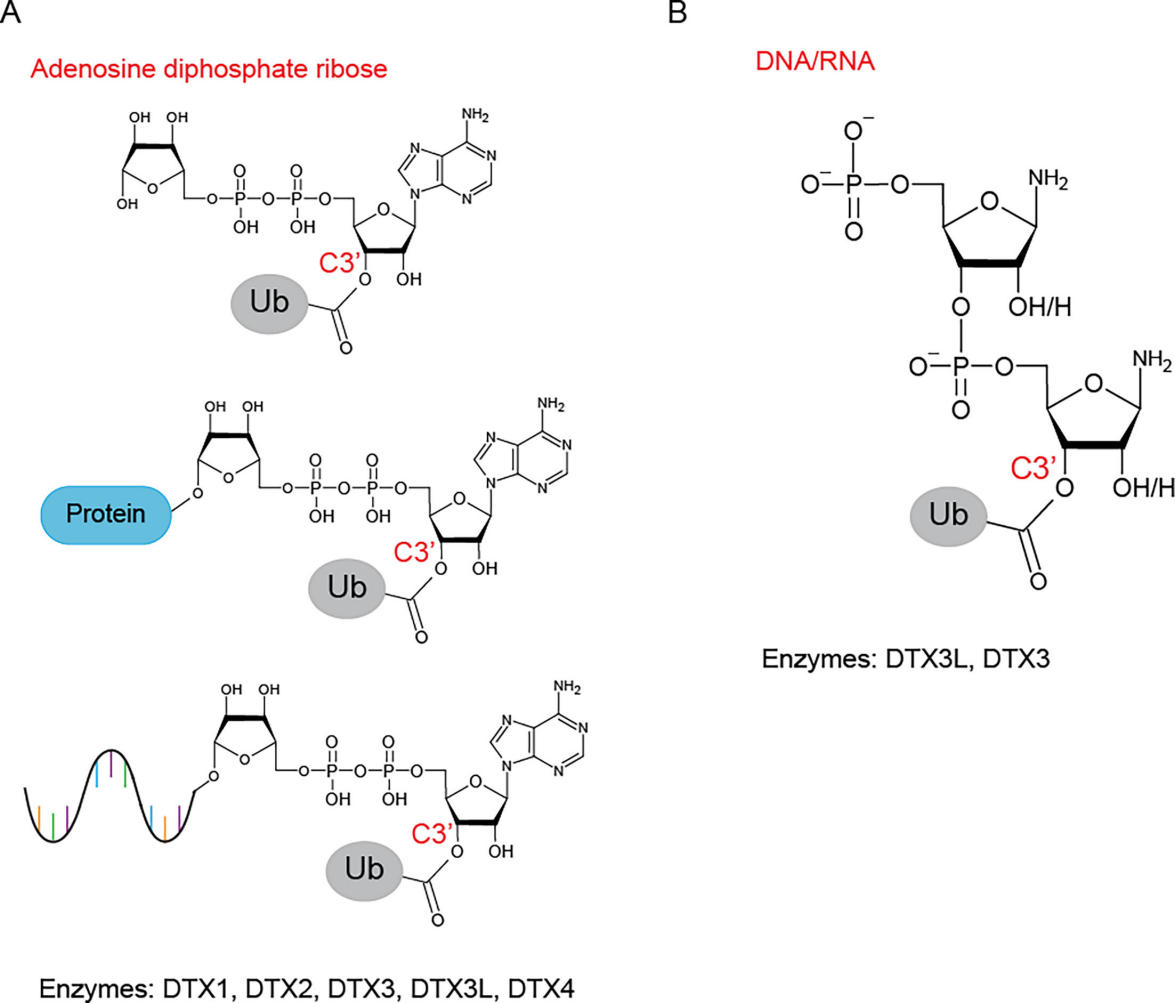
Nucleotides as non-proteinaceous substrates of Ub. (**A**) ADPr is conjugated to Ub by an oxyester bond to the 3′ hydroxyl of the adenine proximal ribose ring. This reaction is catalyzed by all DELTEX family E3s and can occur on free ADPr (top), ADPr moieties on proteins (middle), and ADPr moieties on nucleic acids including DNA and RNA (bottom). (**B**) Only DTX3L and DTX3 from the DELTEX family E3s catalyze the ubiquitination of ssDNA and ssRNA. Ub is conjugated by an oxyester bond to the 3′ hydroxyl of the ribose ring of the 3′ end of the nucleotide. All chemical structures were produced using ChemSketch [7]. ADPr, adenosine diphosphate ribose; ssDNA, single-stranded RNA; ssRNA, single-stranded RNA; Ub, ubiquitin.

Adenosine diphosphate ribose (ADPr) is a PTM catalyzed by the poly-ADP-ribose polymerase family. The discovery of a dual Ub ADPr modification highlighted the potential for cross-talk between PTMs. Initially thought to be catalyzed by PARP9 [28], it was later established that DTX3L, a RING E3 ligase, was responsible for the Ub-ADPr modification [29]. In fact, all members of the DELTEX family, of which DTX3L is a part of, are capable of catalyzing the conjugation of Ub and ADPr [29–31], and the conserved C-terminal RING domain followed by the DELTEX-C-terminal domain (DTC) is the minimally catalytically competent fragment [28,29,31]. Canonical ADP-ribosylation activity involves the release of nicotinamide from NAD^+^ followed by the attachment to the substrate via the C1 atom of the nicotinamide ribose. However, with the Ub-ADPr modification, Gly^76^ of Ub is attached to the 3′-hydroxyl of the adenine proximal ribose ring of ADPr by an oxyester linkage [31] (Figure 2A). As 3′-hydroxyl is present in the adenine ribose of NAD^+^, AMP, and ADP, DELTEX E3s also ubiquitinate these molecules *in vitro* [29,31].

The DTC domain of the DELTEX proteins contains a pocket that binds ADPr and NAD^+^, enabling it to recruit poly-ADPr-modified substrates for ubiquitination of ADPr moieties [29,30]. Therefore, in addition to free ADPr, ADPr moieties on ADPr-modified proteins [31] and nucleic acids [32] can be ubiquitinated. Within the DTC domain pocket of DTX2, His^594^ was identified as important residue for ADPr binding [30]. In addition, two conserved catalytic residues have been identified within the same pocket, His^582^ and Glu^608^ in DTX2, which, along with ADPr, form a linear catalytic triad-like arrangement proposed to activate the 3′-hydroxyl of the adenine ribose for ubiquitination [31]. Structural modeling of E2-conjugated Ub (E2-Ub) onto the structure of DTX2-ADPr complex showed that the flexibility in the RING-DTC domain could bring E2-Ub active site into the proximity of 3′-hydroxyl of the adenine ribose of ADPr, revealing a plausible mechanism of catalysis [29,31].

To date, only the ubiquitination of ADPr moieties on protein has been linked to a biological function. This is a result of the successful characterization of the Ub modification of mono-ADP-ribosylated (MARylated) PARP10 in cells [33]. This relied on a methodology utilizing various enzymes and chemical treatments such as TssM*, a DUB with Ub esterase activity and NUDT16, a hydrolase that cleaves protein MARylation to confirm it is MARylated Glu/Asp sites on PARP10 that are ubiquitinated in cells [33]. The dual PTM (referred to as MARUbylation) is extended with K11-linked Ub chains, which target PARP10 for proteasomal degradation [33], providing functional relevance for this modification.

### Ub modification of nucleic acids

Most recently, nucleic acids have been identified as substrates for ubiquitination. Both single-stranded RNA (ssRNA) and single-stranded DNA (ssDNA) can be ubiquitinated by DTX3L, forming an oxyester linkage between Ub and the 3′-hydroxyl group of the ribose ring on the 3′ nucleotide [34,35] (Figure 2B). Interestingly, only the RING and DTC domains were required for Ub modification of single-stranded nucleic acids [34,35]. ssDNA binds to the same pocket in the DTC domain as ADPr, and the two molecules compete directly for binding [34]. Mutation of Tyr^719^, the corresponding residue to DTX2 His^594^ which is important for ADPr binding [29], reduced the Ub modification of ssDNA. Similarly, the mutation of the corresponding aforementioned catalytic triad residues, His^707^ and Glu^733^ in DTX3L, abolished or impaired ubiquitination of ssDNA [34], suggesting mechanistic similarities between Ub modification of single-stranded nucleic acids and ADPr. One critical question is how DTX3L selects substrates for ubiquitination. When comparing the catalytic efficiency of DTX3L in catalyzing the ubiquitination of ssDNA and NAD^+^, DTX3L displays ~4–5-fold higher catalytic efficiency for ssDNA, primarily due to a lower *K*_m_ value [34]. However, at higher substrate concentrations, DTX3L exhibits a higher rate of Ub modification of NAD^+^ than ssDNA [34]. This indicates that the local concentration of these substrates within cellular microenvironments may regulate DTX3L’s substrate specificity.

Although all members of the DELTEX E3 family catalyze Ub modification of ADPr, only DTX3L and DTX3 have been identified as capable of ubiquitinating nucleic acids [34,35]. Structural predictions suggest that DTX3L and DTX3 possess RRM- and/or KH-like fold domains in their N-terminal regions. Both RRM and KH domains are well-characterized DNA- and RNA-binding motifs. In contrast, DTX1, DTX2, and DTX4 harbor WWE domains in their N-termini, which are known to bind poly-ADPr [36]. While the RRM/KH-like domains are not required for single-stranded nucleic acid ubiquitination, they may contribute to nucleic acids binding and potentially confer substrate specificity, though further investigation is needed. The presence of these domains alone, however, cannot explain the specificity of Ub modification of nucleic acids by DTX3L and DTX3 as the RING-DTC (RD) domains are the minimally catalytically competent fragment. Additionally, the RING domains of DTX1, DTX2, and DTX4 differ from those of DTX3L and DTX3, as the latter contain multiple insertions, making their RING domains longer. Within the DTC domain, near the ADPr/ssDNA-binding pocket, DTX3L and DTX3 also uniquely possess an Ala-Arg motif, which is absent in DTX1, DTX2, and DTX4. Deletion of this motif in DTX3L abolishes ubiquitination of nucleic acids. However, the introduction of this motif into DTX2 does not confer nucleic acid ubiquitination activity [35]. This suggests that while the Ala-Arg motif is essential for efficient ubiquitination of nucleic acids, it is not the sole requirement. Furthermore, Ub modification of ssDNA only occurs when the RD domains are *in cis*. Chimeric constructs of DTX2 RING domain and DTX3L DTC domain can ubiquitinate NAD^+^ but not ssDNA, indicating that the spatial arrangement of DTX3L RD domains is important for Ub modification of nucleic acid [34].

Despite strong *in vitro* evidence supporting Ub modification of nucleic acid and insights into the underlying mechanism, detecting this modification in cellular contexts and elucidating its functional relevance will require significant advancements in tools and methodologies to study it effectively.

### Deubiquitinases

The reversibility of ubiquitination is important for the regulation of Ub signaling. Deubiquitination is catalyzed by deubiquitinating enzymes (DUBs) that remove Ub from substrates in response to environmental changes and stress, thereby altering substrate fate and recycling Ub. There are ~100 DUBs in the human genome that are responsible for the removal of Ub from diverse substrates, and targeting can be through recognition of the substrate or the Ub chain itself. For most non-proteinaceous substrates, little is known about the specific DUBs that cleave their Ub modifications in cells. One well-characterized example is the endosomal DUB, Doa4, which was shown to be responsible for deubiquitination of Ub-PE in yeast cells [6]. Beyond this, many non-proteinaceous Ub substrates lack identified DUBs in cells, and only a few have been studied *in vitro*. Ub-ADPr can be cleaved by human ubiquitin-specific peptidase 2 (USP2) [29,31,32] and SARS-CoV-2 PLpro [31,32]. Similarly, Ub-ssDNA and ssRNA could also be cleaved by USP2 [32,34]; however, USP2 is a nonspecific DUB that removes all polyUb linkage types [37], and it is not possible to conclude whether Ub-ADPr, Ub-ssDNA, or Ub-ssRNA would act as substrates for USP2 in cells. Conversely, the ubiquitinated Ser/Thr residues and N-GlcNAc motifs in Nrf1 displays some resistance to DUB activity both *in vitro* and in cells [17]. This raises questions about how the ubiquitination of these residues and motifs is regulated. It is almost certain that there are enzymes present in cells that remove Ub from non-proteinaceous substrates, and it is possible that they are highly specific to the substrate or perhaps there are DUBs that recognize the oxyester linkages [38] found to attach Ub to some non-proteinaceous substrates.

### Challenges

The majority of non-proteinaceous substrates identified have been characterized *in vitro*, with few being linked to functions in cells. One of the major challenges in this area of research is identifying and isolating ubiquitinated substrates from cellular contexts. Indeed, it is possible that there are specific conditions yet to be identified that are required for these modifications to arise, making their discovery even more challenging. It is also important to highlight that quantity does not necessarily correlate with impact. The Ub system operates in an equilibrium, and mechanisms probably exist to reverse Ub modification of non-proteinaceous substrates. In the case of Ub-ADPr, the presence of the conjugate was limited in cells due to the lability of Ub-ADPr oxyester linkage and cleavage by DUBs, resulting in difficulty in detection. Overexpression of DTX2 was required to generate detectable levels of Ub-ADPr. Tools capable of recognizing these substrates may aid in characterization. The Af1521 macro-domain, an ADPr recognition protein, was utilized to pull down Ub-ADPr, followed by validation that the conjugate contained both moieties [29]. Given that ubiquitinated non-proteinaceous substrates can be generated *in vitro*, it might be possible to develop nanobodies that selectively bind these products to aid detection and validation in cells. This approach has been used successfully to engineer nanobodies that are specific for K48-K63 branched Ub [39] and have the potential to be applied to non-proteinaceous substrates of ubiquitination. However, in cases where the ubiquitinated species is less defined, the development of tools to identify these will be challenging. Advances in mass spectrometry methods combined with metabolomic approaches could assist in further characterization of ubiquitinated non-proteinaceous substrates which will, in turn, aid in understanding their functional relevance.

It is likely that additional, yet uncharacterized, E3s exist that can ubiquitinate non-proteinaceous substrates. However, caution is needed when assessing the potential contribution of non-specific E3 activity to observed ubiquitination. Studies on RNF213 and DELTEX E3s illustrate two distinct approaches for identifying and validating such E3s. For RNF213, the ubiquitinated substrate (LPS) was identified first, followed by investigations to determine the responsible E3. Fractionation of HeLa cell lysates revealed RNF213 as the key E3 catalyzing LPS ubiquitination *in vitro*. Subsequent RNF213 knockout experiments confirmed its role, as cells lacking RNF213 failed to ubiquitinate LPS upon bacterial infection. Further characterization established that RNF213’s RZ domain was responsible for its E3 activity toward LPS [19]. In contrast, studies on DELTEX E3s began with the identification of a conserved RD domain across the DELTEX family. Within this region, the DTC domain binds ADPr, while the RING domain recruits E2-Ub to catalyze the formation of Ub-ADPr conjugate. Structural and mutagenesis analyses of DTX2-ADPr complex helped establish the specificity of this reaction. Additionally, cell-based assays confirmed that DTX2 could catalyze Ub-ADPr conjugation [29]. Together, these studies highlight different strategies for identifying and validating E3s involved in the ubiquitination of non-proteinaceous substrates. However, discovering additional E3s capable of these modifications remains a significant challenge, as their activity cannot be predicted based on sequence alone. One potential approach is to screen E3s *in vitro* for their affinity and activity toward a broad range of non-proteinaceous substrates. However, such screening is inherently low-throughput, and detecting these modifications may require extensive optimization for each individual substrate. In particular, mass spectrometry-based methods for detecting labile oxyester bonds remain underdeveloped. While challenges remain, the growing recognition of non-proteinaceous substrate ubiquitination is already driving, and will continue to drive, the development of innovative strategies and technological advancements, further advancing our ever-expanding understanding of the Ub system.

**Perspectives**

- Numerous non-proteinaceous substrates of ubiquitination have been identified, expanding the ubiquitin (Ub) system.
- Given the diverse nature of these substrates, the role of Ub is likely far more extensive and complex than that of a conventional post-translational modification.
- Future research should prioritize developing tools and technologies that enable a more in-depth investigation in order to decipher the biological relevance of these modifications.

## Abbreviations

ADPr: adenosine diphosphate ribose
Atg8: autophagy-related protein 8
DTC: DELTEX-C-terminal domain
DUB: deubiquitinating enzyme
E1: Ub-activating enzyme
E2: Ub-conjugating enzyme
E3: Ub-ligase
E2-Ub: E2-conjugated Ub
HOIL-1: heme‐oxidized IRP2 ubiquitin ligase‐1
HOIP: HOIL-1-interacting protein
IBR: in-between-RING
LUBAC: linear ubiquitin chain assembly complex
MARylated: mono-ADP-ribosylated
Nrf1: nuclear factor erythroid 2-like 1
PEs: phosphatidylethanolamines
PTM: post-translational modification
RBR: RING-between-RING
RD: RING-DTC
Tul1: transmembrane E3 ubiquitin-protein ligase 1
UBL: ubiquitin-like protein
Ub: ubiquitin
